# Food insecurity and dietary diversity among lactating mothers in the urban municipality in the mountains of Nepal

**DOI:** 10.1371/journal.pone.0227873

**Published:** 2020-01-14

**Authors:** Devendra Raj Singh, Saruna Ghimire, Satya Raj Upadhayay, Sunita Singh, Umesh Ghimire

**Affiliations:** 1 Department of Public Health, Asian College for Advance Studies, Purbanchal University, Satdobato, Lalitpur, Nepal; 2 Southeast Asia Development Actions Network (SADAN), Lalitpur, Nepal; 3 Department of Sociology and Gerontology and Scripps Gerontology Center, Miami University, Oxford, OH, United States of America; 4 Department of Public Health, National Open College, Pokhara University, Sanepa, Lalitpur, Nepal; 5 Central Department of Home Science, Padma Kanya Campus, Tribhuvan University, Kathmandu, Nepal; 6 New ERA, Kathmandu, Nepal; African Population and Health Research Center, KENYA

## Abstract

**Background:**

Adequate nutrition is essential during the lactation period for better maternal and child health outcomes. Although food insecurity and dietary monotony (defined as less diverse diet), two important determinants of undernutrition, are endemic in the rural mountains of Nepal, insufficiently examined and assessed for risk factors in mothers during lactation, a life stage of high nutritional demand. This study aimed to assess the status and factors associated with food insecurity and dietary diversity among lactating mothers residing in the mountains of Nepal.

**Methods:**

A community-based cross-sectional study was conducted in an urban municipality in the mountainous Bajhang District of far-western Nepal. The sampling frame and strategy led to 417 randomly selected lactating mothers. Household Food Insecurity Access Scale (HFIAS) and the tool “Minimum Dietary Diversity for Women” developed by the Food and Agriculture Organization were used to measure food insecurity and dietary diversity, respectively. Additional information on socio-demographics and risk factors were collected. Multivariable logistics regression assessed correlates of study outcomes.

**Results:**

Overall, 54% of the households were food insecure, and over half (53%) of the mothers had low dietary diversity. Food insecurity status (mild food insecurity AOR = 10.12, 95% CI = 4.21–24.34; moderate food insecurity AOR = 8.17, 95% CI = 3.24–20.59, and severe food insecurity AOR = 10.56, 95% CI = 3.92–28.43) were associated with higher odds of dietary monotony. Likewise, participants with lower dietary diversity were 8.5 times more likely to be food insecure than those with higher dietary diversity (AOR = 8.48, 95% CI = 3.76–19.14). The monthly income of the family was positively associated with food insecurity. Participants’ (AOR = 3.92 95%CI = 1.76–8.71) or spouses’ (AOR = 2.90, 95% CI = 1.07–7.85) unemployment was associated with higher odds of being food insecure. Likewise, owning a cultivable land (AOR = 0.49, 95% CI = 0.28–0.84) and participant’s unemployment status (AOR = 5.92, 95% CI = 3.02–11.63), were significantly associated with increased odds of dietary monotony.

**Conclusion:**

The observed food insecurity and poor dietary diversity among lactating mothers, the correlates associated with these outcomes, may help local stakeholders to identify local health needs and subgroups for targeted interventions. Socioeconomically disadvantaged mothers should be specifically targeted for relevant programs and policies.

## Background

Food sufficiency and dietary quality during the lactation period are essential for maternal and child health. Physiologically, the nutritional demand increases during pregnancy and lactation; during these phases, the requirements for both calorie and essential nutrients are higher [1]. However, maternal and child undernutrition is a severe public health problem globally and accounted for 45% of all child deaths in 2011 [2]. Breast milk provides complete nutrition for the healthy growth of an infant [3–5]. The World Health Organization (WHO) recommends exclusive breastfeeding up to six months of a child's age and continued breastfeeding, along with complementary feeding, up to two years of age [6]. The quantity of breast milk largely depends on the nutritional quality and adequacy of the mother’s diet [7]. Thus, it is essential that the diet of a lactating mother is rich in both calorie and nutrients [8].

Despite the higher nutritional requirements, large numbers of lactating mothers, particularly in South Asia, are extremely vulnerable to nutritional deficiencies [9–11]. Their daily diets predominantly include starchy staples with limited or no animal products, fresh fruits, and vegetables [10]. Food insecurity and dietary monotony (defined as less diverse diet) are two important determinants of undernutrition. The Food and Agriculture Organization of the United Nations (FAO) defines food security as “the situation when all people, at all times, have physical, social and economic access to sufficient, safe and nutritious food that meets their dietary needs and food preference for an active and healthy life” [12]. In another term, a household is considered to be food secured when its occupants do not have to live in hunger or fear of starvation [13]. Likewise, FAO defines “minimum dietary diversity” for the reproductive age women as the consumption of at least five of the ten specified food groups through the previous day and night (24 hours period) preceding the survey day [14]. To summarize, food security indicates food adequacy, whereas dietary diversity suggests nutrients adequacy [13,14]. Notably, food adequacy (or enough food to eat) does not necessarily indicate eating diversified food or intake of various essential nutrients. When studied together, food security and dietary diversity provide a comprehensive understanding of nutritional well-being.

Nepal, a low-income country nestled in the Himalaya between India and China, has made remarkable progress in some important health and nutritional targets of the Millennium Development Goals [15], yet, hunger and malnutrition are a common problem among Nepali children and women [16,17]. The country ranks 72^nd^ of the 118 countries in the world in terms of the Global Hunger Index [18], with women and children being most vulnerable to the risk of hunger and malnutrition. A 2016 survey reported 18% of the Nepalese women of the reproductive age to be malnourished [17]. Specifically, 41% were anemic with a higher prevalence (46%) among pregnant and lactating women [17]. Dietary diversity among lactating mother is poor and often includes a large portion of starchy foods. Different types of food habits and dietary taboos, imbedded within different beliefs of diverse ethnic groups in Nepalese society also plays a significant role in limiting the dietary adequacy and diversity of pregnant and lactating mothers [19,20]. As for an example, energy-dense foods are encouraged while fruits, vegetables, and cold foods are discouraged postnatal [21,22].

Nationally, food insecurity among Nepali households ranges between 41%-52%, with 7%-10% of the households severely food insecure [16,17]. Likewise, 51% of the women of reproductive age failed to meet the minimum dietary diversity [16]. Furthermore, both food insecurity and dietary monotony were higher in households from rural, mountainous, and the western region of the country [16,17]. To elaborate, the household food insecurity in rural and mountains were 62% each (urban: 46%; Hill: 54%; Terai: 49%), and mid- and far-western regions were >60% (Eastern and Central regions ~50% each) respectively [17]. Although food insecurity and dietary monotony are endemic in the mountains of Nepal, they are insufficiently examined and assessed for risk factors in mothers during lactation, a life stage of high nutritional demand. Further, the existing geographic inequalities in food insecurity is considered as a major challenge in achieving the second goal of Sustainable Development, i.e., “Zero Hunger,” by 2030 [23]. Policymakers and stakeholders claim a lack of adequate local evidence as one of the barriers to devise and implement programs and policies [24]. Moreover, the recent federalization of Nepal in 2015 handed the administrative powers to the provincial and local governments. To better understand the local health needs and allocate resources accordingly, the de novo local government will need local evidence. Thus, this study aimed to assess the status and factors associated with food insecurity and dietary monotony among lactating mothers residing in the mountains of Nepal.

## Materials and methods

### Study setting

A community-based cross-sectional study was conducted from November-December 2018 among lactating mothers residing in the mountainous Bajhang district in far-western Nepal. Of the twelve municipalities in the district, Jaya Prithivi municipality was randomly selected for the study. According to the most recent census, Jaya Prithivi municipality is one of the largest urban municipalities of the Bajhang district, with a total population of 20,280 (9738 males and 10,542 females) living in 4,015 households [25]. Although the road infrastructure is not well developed in most parts of the district, the district headquarters, i.e., Jaya Prithivi municipality (notably study site), has been recently connected to the country's road network, thus facilitating road access to the study sites [26].

### Study design and sample

The sample size of 417 was calculated by using the formula n = Z^2^pq/d^2^. Here, Z = standard normal deviation and equals 1.96 at 95% confidence level; p is the prevalence of the outcome of interest which was set at 50% [27] considering unknown prevalence of dietary diversity or food security among lactating mothers in the study area; q = 1-p; and the allowable error (d) was set at 5%; and 10% non-response rate was added.

Systematic random sampling was used to select households. The municipality selected for this study has 11 wards (the lowest administrative unit in Nepal), of which six wards (50%) were randomly selected. The number of the household in each of the selected wards was obtained from the Census Bureau [25], and the required number of households and corresponding sampling interval in each ward was calculated based on probability proportional to the size of the ward (defined in terms of the number of households in the ward). In each ward, the first household, in the direction of a pencil tip, was selected by rotating a pencil. Every household in the k^th^ sampling interval was then visited and screened for eligible participants. One eligible mother was selected from each household; therefore, the number of households equals the number of participants in the study. In the case of more than one eligible participant in the same household, one was selected by the lottery method. In the absence of an eligible participant in the selected household, an eligible participant from the adjacent household was sought. Eligibility criteria included: a lactating mother, defined according to WHO’s definition of complementary feeding [6] as a breastfeeding mother of under two (years) child living permanently in the study area.

### Data collection and variables

Data were collected through individual face-to-face interviews at the respondent’s home in the Nepali language. Surveyors and supervisors were public health professionals who were fluent in the local language. Surveyors received three days of training on data collection, sample selection, study tools administration, and data handling procedures. As such, surveyors were familiar with the objectives, methods, and ethical aspects of the study. To ensure the quality of the collected data, field supervisors cross-checked the filled questionnaire on-site, and any discrepancies and/or missing data were re-inquired. Study tools were translated into Nepali language and pretested among ten percent of the study sample (n = 42) in a nearby municipality that was not included in the study. Food insecurity and dietary diversity were the two-outcome variables of interest.

#### Food insecurity

Food insecurity was measured using the Household Food Insecurity Access Scale (HFIAS) developed by USAID’s Food and Nutrition Technical Assistance (FANTA) project [13]. The HFIAS is a cross-culturally validated tool and has also been used in the 2016 Nepal Demographic and Health Survey [13,17]. Details of the HFIAS tool are defined elsewhere [13]. Briefly, the HFIAS consists of nine “frequency-of-occurrence” questions (specified in S1 Table) that measures the severity of food insecurity in the last four weeks in terms of four Likert scale responses [0 = never, 1 = rarely (once or twice), 2 = sometimes (three to ten times), 3 = often (more than ten times)]. The respondents were expected to answer these questions on behalf of all household members. The cumulative score of the nine items ranged between 0–27, and a higher HFIAS score indicated more food insecurity the household experienced (in terms of access to food). The cumulative HFIAS score was categorized into four levels of household food insecurity: food secured, and mild, moderate, and severe food insecurity, following HFIAS guidelines [13]. In our analyses, we combined the three (mild, moderate, and severe) food insecure categories to form a dichotomous outcome variable (food secured and insecure). The Cronbach's alpha for the HFIAS in our study was 0.84, indicating high reliability or internal consistency of the scale.

#### Dietary diversity

The dietary diversity score (DDS) of the lactating mothers was measured by using 24 hours dietary recall following the guidelines on minimum dietary diversity for women (MDD-W) developed by FAO and FANTA Project, and it is comprehensively defined elsewhere [14]. Ten food groups, listed in S2 Table, were included in the DDS. Consequently, the minimum possible DDS is zero (none of the ten food groups were consumed in the past 24 hours), and the maximum is ten (10 food groups were consumed in the past 24 hours). Therefore, the higher the DDS, the greater the diversity in the food intakes, which indicates better nutrient intake among the lactating mothers. The cumulative DDS score was categorized to construct a dichotomized outcome variable: high dietary diversity (consuming five or more food groups) and low dietary diversity or dietary monotony (consuming less than five of the ten defined food groups) based on the recent recommendation by the women’s Dietary Diversity Project Study Group [28].

### Socio-demographic variables

Information on socio-demographic variables such as participants’ age, ethnicity, education, family structure, migration of family members for employment, participants’ and spouses’ employment status, participants’ age at marriage and first pregnancy, and antenatal care (ANC) visits during the last pregnancy were collected from the participants by interview. Study participant’s ethnicity was categorized into three major categories prevalent in Nepali Hill’s society: Bhramin, Chhetri, and Dalits or minorities [29]. Family structure was classified into nuclear (husband and wife living with their children who are under the age of eighteen years) and joint or extended family (husband and wife living with their children who are under the age of eighteen years of age and older parents and relatives in the same household). A binary response question asked if any family member has migrated, for at least six months, within or outside the country for employment or income generation. Socio-economic variables included monthly family income and whether the household possessed a cultivable land or farm. Age at marriage, and subsequently age at pregnancy, was categorized into binary responses (<20 years and ≥20 years) as per the Nepal Civil code [30], which has set 20 years of age as the minimum age of marriage under Nepali law.

### Data processing and statistical analyses

The collected data were entered into EpiData software 3.1v and transferred into IBM SPSS 21 for statistical analyses. The descriptive results are presented in the form of mean, standard deviation, frequency, and percentage. The difference in mean and proportion was measured using independent t-tests and Pearson’s chi-square (χ2), respectively. To assess the correlates, binary logistic regression models were conducted separately for the two dichotomized outcomes: food insecurity and dietary diversity. Independent variables significant at or below 0.2 in the unadjusted models were adjusted for in the adjusted model. Variance inflation factor was <2.5, suggesting multicollinearity was not an issue in the model. The statistical significance was considered at p-value <0.05 and 95% confidence interval (CI).

### Ethical considerations

Ethical clearance for this study was obtained from the Ethical Review Board of Nepal Health Research Council (Ref no: 746/018). The local municipality office also provided consent. Informed written consent was obtained from each of the participants before proceeding with the data collection. Participants were informed about the voluntary participation, their right to refusal at any point, and the confidentiality of their identity.

## Results

### Participants’ demographic and socio-economic characteristics

The mean age of respondents was 25.4 ± 5.0 years (Table 1). Mostly, participants were from Chhetri ethnicity (37%), lived in a joint/extended family (59%) of average size 5.4. The majority of the respondents did not have formal education (36%) and were unemployed (65%). The average (± SD) monthly income of the household was USD 115.02 ± SD 63.79, 59% of the households owned farming land, and 45% of the households had a family member(s) migrated for employment. The mean (± SD) age at marriage and first pregnancy for the respondents was 18 (±2.3) and 20 (±2.2) years, respectively; most of these marriages and pregnancies occurring before age 20. Almost half of the women (48%) were first-time moms. About 30% of the women had less than four ANC visits. (Table 1)

**Table 1:** Socio-demographic characteristics of lactating mothers by household food insecurity and dietary diversity status in Jaya Prithivi Municipality, Bajhang District, Nepal (n = 417).

Variables	Total sample N = 417	Food security status			Dietary diversity status		
		Secure (n = 191, 45.8%)	Insecure (n = 226, 54.2%)	p-value	High (n = 194, 46.5%)	Low (n = 223, 53.5%)	p-value
* Demographic characteristics*	n (%)	n (%)	n (%)		n (%)	n (%)	
**Mean age (± SD)**	25.4 ± 5.0	25.7 ± 4.8	25.1 ± 5.2	0.224^a^	25.2 ± 4.9	25.6 ± 5.2	0.429^a^
**Ethnicity**							
Brahmin	123 (29.5)	80 (41.9)	43 (19.0)	**<0.001**	72 (37.1)	51 (22.9)	**0.005**
Chhetri	156 (37.4)	64 (33.5)	92 (40.7)		68 (35.1)	88 (39.5)	
Dalits and minorities	138 (33.1)	47 (24.6)	91 (40.3)		54 (27.8)	84 (37.7)	
**Family structure**							
Nuclear	173 (41.5)	113 (59.2)	60 (26.5)	**<0.001**	89 (45.9)	84 (37.7)	0.092
Joint/extended	244 (58.5)	78 (40.8)	166 (73.5)		105 (54.1)	139 (62.3)	
**Participants’ education**							
No formal education	151 (36.2)	47 (24.6)	104 (46.0)	**<0.001**	49 (25.3)	102 (45.7)	**<0.001**
Grade 1–5	100 (24.0)	54 (28.3)	42 (18.6)		52 (26.8)	44 (19.7)	
Grade 6–10	113 (27.1)	54 (28.8)	59 (26.1)		58 (29.9)	55 (24.7)	
Grade 10 and above	53 (12.7)	36 (18.8)	21 (9.3)		35 (18.0)	22 (9.9)	
***Economic characteristics***							
**Participants’ employment**							
Employed	147 (35.3)	98 (51.3)	49 (21.7)	**<0.001**	108 (55.7)	39 (17.5)	**<0.001**
Unemployed	270 (64.7)	93 (48.7)	177 (78.3)		86 (44.3)	184 (82.5)	
**Spouses’ employment**							
Employed	280 (67.1)	171 (89.5)	109 (48.2)	**<0.001**	148 (76.3)	132 (59.2)	**0.002**
Unemployed	137 (32.9)	20 (10.5)	117 (51.8)		46 (23.7)	91 (40.8)	
**Owning cultivable land**							
Yes	246 (59.0)	130 (68.1)	116 (51.3)	**<0.001**	138(71.1)	108 (48.4)	**<0.001**
No	171 (41.0)	61 (31.9)	110 (48.7)		56 (28.9)	115 (51.6)	
**Family monthly income**^**b**^ (mean ±SD) USD	115.0 ± 63.8	153.0 ± 58.5	82.8 ± 48.6	**<0.001**^**a**^	133.2 ± 64.4	99.1 ± 58.9	**<0.001**^**a**^
**Family member migrated for employment**							
No	229 (54.9)	59 (30.9)	170 (75.2)	**<0.001**	89 (45.9)	140 (62.8)	**0.002**
Yes	188 (45.1)	132 (69.1)	56 (24.8)		105 (54.1)	83 (37.2)	
***Reproductive characteristics***							
**Age at marriage (mean ± SD)**	18.1 ± 2.3	18.6 ± 2.3	17.6 ± 2.3	**<0.001**^**a**^	18.4 ± 2.5	17.8 ± 2.2	**0.023**^**a**^
**Age at marriage category**							
<20 years	293 (70.3)	105 (55.0)	188 (83.2)	**<0.001**	126 (64.9)	167 (74.9)	**0.027**
≥20 years	124 (29.7)	86 (45.0)	38 (16.8)		68 (35.1)	56 (25.1)	
**Age at first pregnancy (mean ± SD)**	19.7 ± 2.2	20.2 ± 2.1	19.3 ± 2.2	**<0.001**^**a**^	20.0 ± 2.3	19.4 ± 2.1	**0.008**^**a**^
**Age at first pregnancy**							
<20 years	187 (45.0)	60 (31.6)	127 (56.2)	**<0.001**	73 (37.6)	114 (51.4)	**0.003**
≥20 years	229 (55.0)	130 (68.4)	99 (43.8)		121 (62.4)	108 (48.6)	
**Parity**							
Primipara	201 (48.2)	93 (48.7)	108 (47.8)	0.922	106 (54.6)	95 (42.6)	**0.009**
Multiparous	216 (51.8)	98 (51.3)	118 (52.2)		88 (45.4)	128 (57.4)	
**ANC check-up during last pregnancy**							
<4 times	124 (29.7)	6 (3.1)	118 (52.2)	**<0.001**	19 (9.8)	105(47.1)	**<0.001**
≥4 times	293 (70.3)	185 (96.9)	108 (47.8)		175 (90.2)	118 (52.9)	

^a^: p-value from independent t-test; all others are from the Chi-Square test. Significant p-values are bolded.

^b^: USD $1 = NRs.100 (Nepalese currency); SD: standard deviation.

### Prevalence of food insecurity and low dietary diversity

Participants’ responses to the nine items of HFIAS are provided in S1 Table. More than half (54%) of the households were food insecure (mild = 22.5%, moderate = 16.8%, severe = 14.9%) (Table 1 and S1 Table).

Likewise, over half (53%) of the women had low dietary diversity (Table 1). Participants’ 24-hours consumption of each of the ten food groups included in the dietary diversity score is provided in S2 Table. Of the ten food groups, consumption of meat, fish, poultry, and eggs were concerningly low (S2 Table).

### Correlates of food insecurity

In unadjusted analyses, participants’ ethnicity, family structure, education, participants’ and their spouses’ employment, household income, and economic characteristics, as well as women’s reproductive characteristics, were associated with food insecurity (Table 2). In the multivariable logistic regression (Table 2), participants’ ethnicity, family structure, age at marriage, ANC visits, and economic indicators remained statistically significant after adjusting for covariates. Compared to the Brahmins, participants from Chhetri (AOR = 2.85, 95% CI = 1.23–6.62) were 185% more likely to be food insecure. On the other hand, living in a nuclear family structure (AOR = 0.13, 95% CI = 0.06–0.29) were associated with 87% lower odds of food insecurity. Participant’s early age at marriage (< 20 years) and receiving less than the recommended ANC visits were significantly associated with higher odds of being food insecure.

**Table 2:** Unadjusted and adjusted odds ratios using logistic regression for factors associated with food insecurity among lactating mothers in Jaya Prithivi Municipality, Bajhang District, Nepal (n = 417).

	Food insecurity	
	Unadjusted	Adjusted ^a^
	OR (95%CI)	OR (95%CI)
***Demographic characteristics***		
**Age of participants**	0.97 (0.94–1.01)	0.94 (0.85–1.05)
**Ethnicity**		
Brahmin	*Reference*	*Reference*
Chhetri	**2.67 (1.64–4.36)**	**2.85 (1.23–6.62)**
Dalit and minorities	**3.6 (2.16–6.00)**	1.74 (0.66–4.61)
**Family structure**		** **
Nuclear	**0.24 (0.16–0.37)**	**0.13 (0.06–0.29)**
Joint/extended	*Reference*	*Reference*
**Participants’ education**		
No formal education	**3.79 (2.00–7.18)**	0.64 (0.17–2.36)
Primary education (Grade 1–5)	1.33 (0.68–2.61)	0.48 (0.13–1.80)
Secondary education (Grade 6–10)	1.87 (0.97–3.59)	0.73 (0.21–2.58)
Grade 10 and above	*Reference*	*Reference*
***Economic characteristics***		
**Participants’ employment**		
Employed	*Reference*	*Reference*
Unemployed	**3.80 (2.48–5.82)**	**3.92 (1.76–8.71)**
**Spouses’ employment**		
Employment	*Reference*	*Reference*
Unemployment	**9.17 (5.39–15.61)**	**2.90 (1.07–7.85)**
**Owning cultivable land**		
No	*Reference*	*Reference*
Yes	**0.49 (0.33–0.73)**	1.00 (0.48–2.12)
**Family monthly income**		
Tertile 1 = lowest	**135.76 (48.99–376.23)**	**71.71 (14.28–360.15)**
Tertile 2	**7.08 (3.64–13.78)**	**9.17 (2.94–28.58)**
Tertile 3	*Reference*	*Reference*
**Family member migrated for employment**		
No	*Reference*	*Reference*
Yes	**0.14 (0.09–0.22)**	0.63 (0.23–1.72)
***Reproductive characteristics***		
**Age at marriage**		
<20 years	**4.00 (2.55–6.29)**	**3.92 (1.54–9.96)**
≥20 years	*Reference*	*Reference*
**Age at first pregnancy**		
<20 years	**2.77 (1.85–4.16)**	0.87 (0.37–2.06)
≥20 years	*Reference*	*Reference*
**Parity**		
Primipara	*Reference*	*Reference *
Multiparous	1.03 (0.70–1.52)	1.52 (0.54–4.29)
**ANC check-up during last pregnancy**		
<4 times	*Reference*	*Reference*
≥4 times	**0.03 (0.01–0.07)**	**0.06 (0.02–0.20)**
**Dietary diversity**		
High	*Reference*	*Reference*
Low	**10.22 (6.51–16.06)**	**8.48 (3.76–19.14)**

Significant odds ratio is bolded; OR: Odds Ratio; CI: Confidence Interval; ANC: Antenatal care

^a^ A single model was run adjusting for the variables significant at or below 0.2 in the unadjusted model; included all variables shown in this table.

The monthly income of the family was positively associated with food insecurity; participants at the lower-income tertiles had significantly higher odds of food insecurity than those at the highest tertile. Participants’ (AOR = 3.92 95%CI = 1.76–8.71) or spouses’ (AOR = 2.90, 95% CI = 1.07–7.85) unemployment was associated with four- and three-times higher odds of being food insecure, respectively (Table 2). Participants with lower dietary diversity were 8.5 times more likely to be food insecure than those with higher dietary diversity (AOR = 8.48, 95% CI = 3.76–19.14).

### Correlates of low dietary diversity

In unadjusted analyses, participants’ ethnicity, education, participants’ and spouses’ employment, household income, and owning a cultivable land, as well as women’s reproductive characteristics, were associated with low dietary diversity (Table 3). In the multivariable logistic regression (Table 3), owning a cultivable land was associated with 51% reduced odds of dietary monotony (AOR = 0.49, 95% CI = 0.28–0.84). Unemployed participants were almost six times more likely to have dietary monotony than those employed (AOR = 5.92, 95% CI = 3.02–11.63). Participants who had received the recommended ANC visits (AOR = 0.36, 95% CI = 0.18–0.71) were 64% less likely to have dietary monotony than their counterparts. Food insecurity status (mild food insecurity AOR = 10.12, 95% CI = 4.21–24.34; moderate food insecurity AOR = 8.17, 95% CI = 3.24–20.59, and severe food insecurity AOR = 10.56, 95% CI = 3.92–28.43) were associated with higher odds of dietary monotony (Table 3).

**Table 3:** Unadjusted and adjusted odds ratios using logistic regression for factors associated with low dietary diversity among lactating mothers in Jaya Prithivi Municipality, Bajhang District, Nepal (n = 417).

	Low dietary diversity	
	Unadjusted	Adjusted ^a^
	OR (95%CI)	OR (95%CI)
***Demographic characteristics***	** **	** **
**Age of participants**	1.01 (0.97–1.05)	0.95 (0.88–1.02)
**Ethnicity**		
Brahmin	*Reference*	*Reference*
Chhetri	**1.86 (1.15–3.01)**	1.19 (0.63–2.27)
Dalit and minorities	**2.24 (1.36–3.68)**	0.88 (0.43–1.78)
**Family structure**		
Nuclear	0.70 (0.48–1.04)	1.65 (0.91–3.01)
Joint/extended	*Reference*	*Reference*
**Participants’ education**		
No formal education	**3.28 (1.74–6.18)**	0.64 (0.24–1.72)
Primary education (Grade 1–5)	1.35 (0.69–2.62)	**0.31 (0.11–0.89)**
Secondary education (Grade 6–10)	1.51 (0.79–2.89)	0.39 (0.14–1.03)
Grade 10 and above	*Reference*	*Reference*
***Economic characteristics***		
**Participants’ employment**		
Employed	*Reference*	*Reference*
Unemployed	**5.89 (3.77–9.21)**	**5.92 (3.02–11.63)**
**Spouses’ employment**		
Employment	*Reference*	*Reference*
Unemployment	**2.23 (1.46–3.42)**	0.82 (0.39–1.74)
**Owning cultivable land**		
No	*Reference*	*Reference*
Yes	**0.38 (0.25–0.57)**	**0.49 (0.28–0.84)**
**Family monthly income**		
Tertile 1 = lowest	**6.16 (3.41–11.13)**	1.30 (0.45–3.79)
Tertile 2	**2.03 (1.22–3.37)**	0.85 (0.42–1.74)
Tertile 3	*Reference*	*Reference*
**Family member migrated for employment**		
No	*Reference*	*Reference*
Yes	**0.51 (0.34–0.75)**	1.53 (0.69–3.37)
***Reproductive characteristics***		
**Age at marriage**		
<20 years	**1.64 (1.07–2.50)**	0.52 (0.25–1.11)
≥20 years	*Reference*	*Reference*
**Age at first pregnancy**		
<20 years	**1.75 (1.18–2.59)**	1.14 (0.61–2.15)
≥20 years	*Reference*	*Reference*
**Parity**		
Primipara	*Reference*	*Reference*
Multiparous	**1.61 (1.09–2.37)**	**2.12 (1.05–4.27)**
**ANC check-up during last pregnancy**		
<4 times	*Reference*	*Reference*
≥4 times	**0.12 (0.07–0.21)**	**0.36 (0.18–0.71)**
**Food insecurity**		
Secure	*Reference*	*Reference*
Mild insecurity	**8.40 (4.78–14.76)**	**10.12 (4.21–24.34)**
Moderate insecurity	**11.16 (5.77–21.57)**	**8.17 (3.24–20.59)**
Severe insecurity	**14.11 (6.80–29.27)**	**10.56 (3.92–28.43)**

Significant odds ratio are bolded; OR: Odds Ratio; CI: Confidence Interval; ANC: Antenatal care

^a^ A single model was run adjusting for the variables significant at or below 0.2 in the unadjusted model; included all variables shown in this table.

## Discussion

This study was conducted to examine the status and factors associated with food insecurity and dietary diversity among lactating mothers residing in the mountains of the far-western Nepal. Given the importance of adequate food and nutrition for the overall well-being of lactating mothers and her breastfed infants [8], the findings, although expected, was concerning since both food security and dietary diversity was low among the study participants. Participants’ socio-economic characteristics were associated with both food security and dietary diversity.

More than half of the women experienced mild to severe food insecurity (54%) and low dietary diversity (53.5%) in their diet. Our estimates are comparable to the findings from the 2016 NDHS, a nationally representative survey, where 53.2% of households in hilly regions of Nepal were food insecure, and 58% of mothers with children aged 6–23 months had an unacceptable level of dietary diversity [17]. Previous studies from India and Bangladesh also report high food insecurity and poor dietary diversity among lactating mothers and/or women of reproductive age [31,32]. In contrast, our findings are comparatively higher than the estimates from the 2016 Nepal National Micronutrient Status Survey, where only 38.6% of the households in the hilly region experienced food insecurity [16]. In subsistence agricultural economy, such as Nepal, seasonal variations in food production, availability, and consumption is common. As a consequence, the Nepalese diet may be vulnerable to substantial changes throughout the year, depending upon the seasonal variability in crop production, and the costs of food in local markets [33]. Therefore, the difference observed between studies could be possibly explained by the data collection period, which can affect the seasonal availability of food in the household. Relatedly, the data collection period did not overlap with the peak harvest period when food is plenty, usually during summer in the study area. Therefore, the seasonality may have overestimated our findings.

High food insecurity and poor dietary diversity noted in this study were expected given that Nepal overall, and Bajhang specifically, has high poverty [34,35]. Specifically, the study district, Bajhang, is one of the districts with the highest human poverty index values and the lowest human development index in the country [35]. Although this study was conducted in an urban municipality, notably, it was a rural area until 2014 and was connected by road to other parts of the country only after 2007 [26,36]. Such socio-economic impoverishment directly correlates with deprivations in nutrition. Particularly among women in the study areas, high prevalence of illiteracy, gender based-inequality, and lack of opportunities for decision making may further increase their vulnerability to inadequate food and nutrition [35,37]. These factors also explain the observed positive association between food insecurity status and dietary monotony. Household food security often determines the intake of diversified diets [38] and was reported in previous studies, too [32,39,40]. In fact, some studies also argue that dietary diversity can be used as a proxy for household food security status [39,41]. Particularly, the household wealth and/or poverty, which has been reported to be a strong predictor of both access to enough and diverse food, may explain the positive association between the two given that the study district is one of the least developed districts in the country [42]. With financial constraints, the households may purchase staples rather than a diverse diet. Better household economy, as indicated by higher household income, employment, and possession of lands, may enhance households’ capacity to afford frequent and diverse diets. Future studies have the opportunity to explore the hypothetical mediating and/or moderating role of household income on the proposed pathway between food security and dietary diversity.

The association between ethnicity and food insecurity was also not surprising and is consistent with previous studies from Nepal [43,44] conducted over an extended period from September 2011 to August 2012 [43] where Dalit, Janjatis, and minorities were more food insecure or had poor nutritional status than Brahmins. Globally, and in the Nepalese context too, a significant relationship between ethnicity and food security exists where the lower ethnic groups or the minority groups are the most vulnerable [45,46]. In general, the minority ethnic groups have low socioeconomic status, limited access to health, education, and employment, and high poverty [47–49], which directly impacts their ability to access food.

A small family, as indicated by a nuclear family structure, was associated with lower food insecurity. A previous study from Nepal and India also found a similar positive relationship between small family size and food security [31]. In a joint or extended family type, where many generations of a family live together, the competing demands to feed many family members may act as a barrier to food access [22,50]. Especially in the Nepalese context where the dependency ratio, an indicator of non-working or dependent population (age 0–14 years and ≥65 years) compared to the working-age population (15–65 years), is as high as 67.2% [51], only one or two family member are economically active in income generation and needs to take care of the daily needs of the remaining members [52].

Better household economy, as indicated by higher monthly family income, owning a cultivable land, and participants’ or spouses’ employment, appeared to have a protective effect on food security and/or dietary diversity. The links between low-income status and food insecurity, in both resource rich and poor settings, are well-documented [53,54]. Higher household income is associated with better health and nutrition outcomes [54]. Employment generates income, which increases the purchasing capacity and enables access to food, health care, and other necessities. With a limited economy, households may not have the privilege to enjoy adequate and more frequent meals [48,55,56]. In Nepal, agriculture is mostly subsistence-based, and thus owning farmland means better access to diversified food since the produced food is used to meet the family needs first. A previous study from Bangladesh also revealed a similar positive relationship between owning farmland and high dietary diversity [57].

Better economic indicators are positively associated with dietary diversity [58]. In a previous study from Nepal [59], dietary diversity showed a linear relationship with household wealth.

Another notable finding is the positive association of food security and dietary diversity with adequate antenatal care; mothers who had received recommended ANC visits (≥4 visits) during the last pregnancy had higher food security and greater dietary diversity. The findings are in line with previous studies from Bangladesh and Nepal [60,61]. The findings may be attributed to the nutritional counseling provided to mothers during their ANC visits. Mothers who are frequently exposed to nutritional counseling services, usually provided by nursing staff or other qualified health professionals at the health facility during the ANC/PNC visits, have better chances of improving their nutritional intake [61,62]. On the other side, mothers who were married during their teenage were more likely to be food insecure. A study conducted among pregnant women in Iran also reported high food insecurity among young pregnant women [63]. Early age at marriage is common in Nepal; 40% of the girls are married before their 18^th^ birthday [64]. Early marriage impacts women’s education, decision-making, and empowerment and, consequently, make them more prone to food insecurity and malnutrition [65].

## Limitation of the study

This study has some limitations. First, the cross-sectional study design inhibits any causal inferences between the outcome (food insecurity and dietary diversity) and the correlates. Data on body mass index of mothers was not obtained, which limits the explicit linkage of dietary diversity with maternal health and nutrition status. Prospective studies in the future may provide more insight into the possible relationships between the outcome and correlates. Lactating women from an urban municipality in the mountains of far-western Nepal were included. Given that geographical inequalities in both food insecurity and dietary monotony, by rurality and ecological zones, has been reported [16,17], the findings cannot be generalized to the lactating mothers from rural areas, or other ecological zones. Although the self-reported nature and 24 hours recall of data collection makes the information prone to social desirability bias and recall bias, often underestimating the strength of the association with food insecurity and dietary diversity, nevertheless, self-report and dietary recall is a very common and effective means of collecting data in epidemiological studies especially in resource-limited settings [66,67]. Specifically, the bias in the estimation of household income and/or remittance is anticipated because the interviewee mothers may not have true knowledge of family wealth in patriarchal households as in Nepal. The measure of dietary diversity is based on 24-hours food consumptions only and thus may be subjected to normal day-to-day variability, and it also does not take into consideration the quantities of food consumed. Nevertheless, the approach used in this study has been widely used and validated as a proxy indicator to capture the diversity; specifically, micronutrient adequacy of women’s diet because women who meet the minimum dietary diversity are more likely to have higher micronutrient intakes [13]. These limitations noted this is one of the pioneering studies highlighting the issues faced by lactating mothers in the mountains of Nepal because previous studies have focused on household food insecurity among general Nepali households or women of reproductive age and children.

## Conclusions

Given that adequate nutrition is essential for the overall well-being of both the mother and her breastfed child, the high prevalence of food insecurity and low dietary diversity among lactating mothers needs urgent attention from the relevant stakeholders. If the government’s commitment to achieving the “Zero Hunger” goal of Sustainable Development by 2030 is to be attained, relevant stakeholders should be cognizant of high food insecurity faced by lactating mothers in the mountains of Nepal and prioritize this issue in the existing policies on maternal and child health. The correlates associated with these outcomes may help local stakeholders to identify local health needs and subgroups for targeted interventions. Specifically, socioeconomically disadvantaged mothers should be targeted for relevant programs and policies. Nutritional programs should target both increasing access to food as well as different nutrients.

## Supporting information

S1 File Raw data set. (SAV)

S1 Table Participants’ responses to the nine items of Household Food Insecurity Access Scale (HFIAS) (n = 417). (DOCX)

S2 Table Food groups included in the dietary diversity score and the frequency by household food insecurity status (n = 417). (DOCX)

## Abbreviations

ANC: Antenatal Care
AOR: Adjusted Odds Ratio
CI: Confidence Interval
DDS: Dietary Diversity Score
FANTA: Food and Nutrition Technical Assistance
FAO: Food and Agriculture Organization
HFIAS: Household Food Insecurity Access Scale
MDD-W: Minimum Dietary Diversity-Women
NDHS: Nepal Demographic and Health Survey
USAID: United States Agency for International Development
