# Supplementary material for: Food insecurity and dietary diversity among lactating mothers in the urban municipality in the mountains of Nepal
Source: PLoS One. 2020 Jan 14;15(1):e0227873. doi: 10.1371/journal.pone.0227873 (PMC6959598; doi:10.1371/journal.pone.0227873)
Supplement: S1 Table — (DOCX) [file pone.0227873.s002.docx]

**S1 Table. Participants’ response to the nine items of Household Food Insecurity Access Scale (HFIAS) (n=417)**

| **Household Food Insecurity Access Scale (HFIAS) items** | **Never**  **n (%)** | **Rarely**  **n (%)** | **Sometimes**  **n (%)** | **Often**  **n (%)** |
| --- | --- | --- | --- | --- |
| Q1. Did you worry that your household would not have enough food? | 134 (32.1) | 123 (29.5) | 114 (27.3) | 46 (11.0) |
| Q2. Did the food that your family bought run out, and you didn't have money to get more? | 194 (46.5) | 164 (39.3) | 30 (7.2) | 29 (7.0) |
| Q3. Did your meals only include a few kinds of cheap foods because your family was running out of money to buy food? | 255 (61.2) | 82 (19.7) | 58 (13.9) | 22 (5.3) |
| Q4. How often were you not able to eat a balanced meal because your family didn't have enough money? | 342 (82.0) | 74 (17.7) | 1 (0.2) | 0 (0.0) |
| Q5. Did you have to eat less because your family didn't have enough money to buy food? | 377 (90.4) | 23 (5.5) | 17 (4.1) | 0 (0.0) |
| Q6. Has the size of your meals been cut because your family didn't have enough money for food? | 345 (82.7) | 48 (11.5) | 24 (5.8) | 0 (0.0) |
| Q7. Did you have to skip a meal because your family didn't have enough money for food? | 385 (92.3) | 17 (4.1) | 15 (3.6) | 0 (0.0) |
| Q8. Were you hungry but didn't eat because your family didn't have enough food? | 389 (93.3) | 13 (3.1) | 15 (3.6) | 0 (0.0) |
| Q9. Did you not eat for a whole day because your family didn't have enough money for food? | 389 (93.3) | 13 (3.1) | 15 (3.6) | 0 (0.0) |
| **Total HFIAS score categorized into food insecurity status** | **n (%)** | | | |
| Food secure | 191 (45.8) | | | |
| Mild food insecurity | 94 (22.5) | | | |
| Moderate food insecurity | 70 (16.8) | | | |
| Severe food insecurity | 62 (14.9) | | | |
