# Supplementary material for: Food insecurity and dietary diversity among lactating mothers in the urban municipality in the mountains of Nepal
Source: PLoS One. 2020 Jan 14;15(1):e0227873. doi: 10.1371/journal.pone.0227873 (PMC6959598; doi:10.1371/journal.pone.0227873)
Supplement: S2 Table — (DOCX) [file pone.0227873.s003.docx]

**S2 Table. Food groups included in the dietary diversity score and the frequency by household food insecurity status (n=417)**

| **Food groups included in dietary diversity score** | **Overall** | **Household food insecurity** | | |
| --- | --- | --- | --- | --- |
|  |  | **Food secure** | **Food insecure** | **p-value^1^** |
|  | **n (%)** | **n (%)** | **n (%)** |  |
| Grains, White roots and tubers, and plantains | 417 (100.0) | 191 (100.0) | 226 (100.0) | - |
| Pulses, beans, peas and lentils | 296 (71.0) | 163 (85.3) | 133 (58.8) | <0.001 |
| Nuts and seeds | 320 (76.7) | 168 (88.0) | 152 (67.3) | <0.001 |
| Dairy | 223 (53.5) | 144 (75.4) | 79 (35.0) | <0.001 |
| Meat, poultry and fish | 68 (16.3) | 43 (22.5) | 25 (11.1) | <0.002 |
| Eggs | 29 (7.0) | 22 (11.5) | 7 (3.1) | 0.001 |
| Dark green leafy vegetables | 233 (55.9) | 141 (73.8) | 92 (40.7) | <0.001 |
| Vitamin A rich fruits and vegetables | 194 (46.5) | 134 (70.2) | 60 (26.5) | <0.001 |
| Other Vegetables | 377 (90.4) | 178 (93.2) | 299 (88.1) | 0.095 |
| Other Fruits | 72 (17.3) | 55 (28.8) | 17 (7.5) | <0.001 |
| **Dietary diversity score dichotomized** |  |  |  |  |
| Low dietary diversity (<5 food groups consumption) | 223 (53.5) | 48 (25.1) | 175 (77.4) | <0.001 |
| High dietary diversity (>5 food groups consumption) | 194 (46.5) | 143 (74.9) | 51 (22.6) |  |
| ^1^p-value from chi-square test. | | | | |
